# Echinatin suppresses esophageal cancer tumor growth and invasion through inducing AKT/mTOR-dependent autophagy and apoptosis

**DOI:** 10.1038/s41419-020-2730-7

**Published:** 2020-07-13

**Authors:** Pan Hong, Qin-Wen Liu, Yao Xie, Qi-Hua Zhang, Long Liao, Qing-Yu He, Bin Li, Wen Wen Xu

**Affiliations:** 1 MOE Key Laboratory of Tumor Molecular Biology and Key Laboratory of Functional Protein Research of Guangdong Higher Education Institutes, Institute of Life and Health Engineering, Jinan University, Guangzhou, China; 2 MOE Key Laboratory of Tumor Molecular Biology and Guangdong Provincial Key Laboratory of Bioengineering Medicine, National Engineering Research Center of Genetic Medicine, Institute of Biomedicine, College of Life Science and Technology, Jinan University, Guangzhou, China

**Keywords:** Cancer therapy, Oesophageal cancer, Autophagy

## Abstract

Esophageal squamous cell carcinoma (ESCC) is one of the most common malignant tumors with poor survival. It is urgent to search for new efficient drugs with good stability and safety for clinical therapy. This study aims to identify potential anticancer drugs from a compound library consisting of 429 natural products. Echinatin, a compound isolated from the Chinese herb *Glycyrrhiza uralensis Fisch*, was found to markedly induce apoptosis and inhibit proliferation and colony-formation ability in ESCC. Confocal fluorescence microscopy data showed that echinatin significantly induced autophagy in ESCC cells, and autophagy inhibitor bafilomycinA1 attenuated the suppressive effects of echinatin on cell viability and apoptosis. Mechanistically, RNA sequencing coupled with bioinformatics analysis and a series of functional assays revealed that echinatin induced apoptosis and autophagy through inactivation of AKT/mTOR signaling pathway, whereas constitutive activation of AKT significantly abrogated these effects. Furthermore, we demonstrated that echinatin had a significant antitumor effect in the tumor xenograft model and markedly suppressed cell migration and invasion abilities of ESCC cells in a dose-dependent manner. Our findings provide the first evidence that echinatin could be a novel therapeutic strategy for treating ESCC.

## Introduction

Esophageal cancer is one of the most common malignant tumors, the mortality of which ranks 6th around the world with poor overall survival^1^. Esophageal cancer has two major histologic subtypes, esophageal squamous cell carcinoma (ESCC) and esophageal adenocarcinoma^2^, and ESCC is the main histologic subtype with ~90% of cases in Asia-Pacific region including China. Failure of chemotherapy and radiotherapy leads to tumor recurrence and poor prognosis mainly due to limited efficiency and side effects, for example, conventional chemotherapeutics target both cancer cells and normal cells resulting in toxic effects on the body^3,4^. Currently, esophagectomy remains the chief strategy for treatment of esophageal cancer^5^, therefore, it has become a top priority to find more stable, effective and safe anticancer drugs.

Natural product is an important resource of new drug candidates, especially for anticancer drugs^6–8^. ~80–83% of the approved anticancer drugs were naturally occurring agents or mimicked natural products^9,10^. As bioactive components from natural products, vincristine, irinotecan, etoposide, and paclitaxel have been historically used in clinical cancer treatment^11–14^. By screening novel chemotherapeutics from natural products, we identified echinatin as an effective anticancer drug candidate from a natural product-based library consisting of 429 compounds. Echinatin is an active component of licorice, which is derived from the roots and rhizomes of *Glycyrrhiza* species (Leguminosae) and related species^15^. Licorice extract has many biological effects, including antioxidant, anti-inflammatory, and anticancer, partly through regulation of Nrf2, NO, and NFĸB pathways^16,17^. However, up to now, the biological function of echinatin in cancer treatment has not been reported, and the molecular mechanism remains unknown to a large extent.

Autophagy plays a dual role in tumorigenesis, although some studies reported that autophagy exerts pro-survival effect, most agents-induced autophagy mainly leads to cell death in cancer^18,19^. Autophagy can be regulated by many signaling pathways including PI3K/AKT/mTOR pathways, which is crucial in tumor initiation and progression. Our previous studies found that activation of PI3K/AKT signaling plays a vital role in ESCC growth and metastasis^20,21^. Therefore, novel therapeutic strategies that target autophagy and AKT/mTOR signaling are urgently needed. In this study, we examined the effects of echinatin on growth, chemosensitivity, and metastatic potential of ESCC cells in vitro and in vivo. Moreover, RNA sequencing (RNA-seq), ingenuity pathway analysis (IPA), and a series of functional assays were performed to investigate whether echinatin exerts the anticancer effects through regulation of autophagy and AKT/mTOR pathways.

## Results

### Echinatin induces apoptosis and decreases cell proliferation in ESCC

**Fig. 1:**
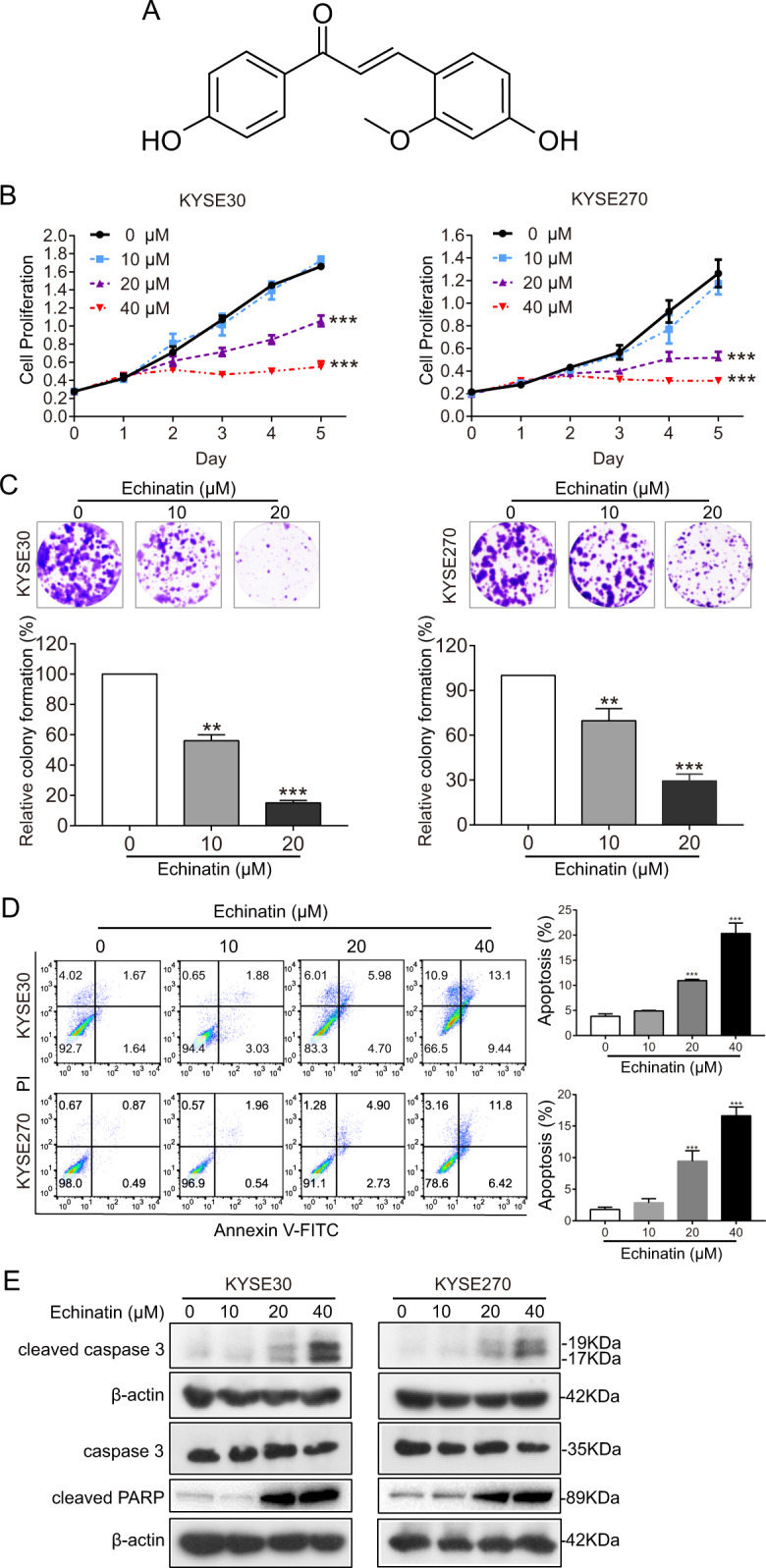
Echinatin inhibits ESCC cell proliferation. **a** Chemical structure of echinatin. **b** KYSE30 and KYSE270 cells were treated with echinatin at different concentrations (for up to 40 μM), and cell viability determined by CCK-8 assay. **c** The colony formation of KYSE30 and KYSE270 cells was inhibited upon exposure to echinatin. **d** The detection of apoptosis in ESCC cells treated with different concentrations of echinatin for 48 h by an Annexin V-FITC/PI double staining assay. **e** Western blot analysis was performed to detect the expression of cleaved caspase-3, caspase-3, and cleaved PARP in the ESCC cells treated with indicated concentrations of echinatin for 48 h. Bars, SD; **P < 0. 01, ***P < 0.001.

We have identified effective anticancer agents from a food-source natural product library consisting of 429 compounds previously^22^. In this study, echinatin, an active component of licorice derived from the roots and rhizomes of *Glycyrrhiza* species (Leguminosae)^15^ (Fig. 1a), was identified as a candidate agent in inhibiting ESCC cell proliferation. To evaluate the effect of echinatin on ESCC cell proliferation, KYSE30 and KYSE270 cells were treated with increasing concentrations of echinatin for up to 5 days. As shown in Fig. 1b, echinatin inhibited cell proliferation substantially in both dose-and time-dependent manner. The colony-formation assay indicated that echinatin markedly declined the number of colony formation (Fig. 1c). Collectively, the above results demonstrated that echinatin might exhibit tumor-suppressive bioactivity in ESCC. To determine the effect of echinatin on apoptosis in ESCC cells, the cells were treated with echinatin at the indicated concentrations (up to 40 μM) for 24 and 48 h, and the results showed that echinatin-induced apoptosis in a dose-dependent way (Figs. 1d and S1A), indicating that echinatin elicited apoptosis in ESCC cells. The induction of apoptosis by echinatin was further evidenced by the increase of apoptotic markers, including cleaved caspase-3 and cleaved poly ADP ribose polymerase (PARP), upon echinatin treatment (Figs. 1e and S1B). Taken together, these data suggest that echinatin can trigger apoptosis to repress proliferation of ESCC cells.

### Echinatin induces autophagy in ESCC cells

**Fig. 2:**
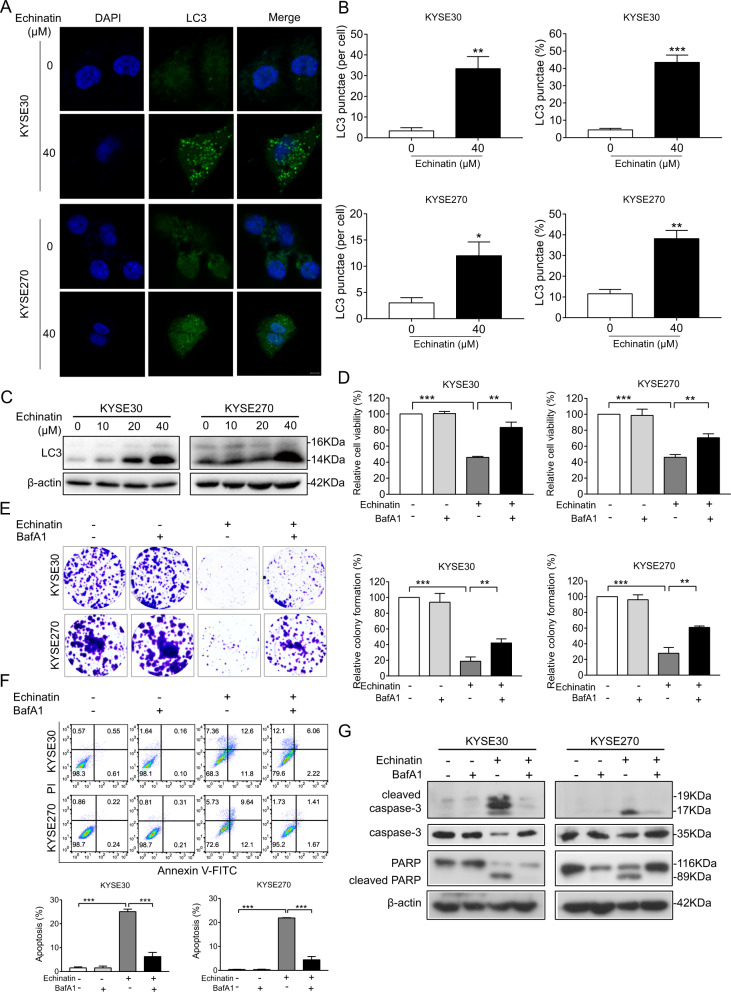
Echinatin induces autophagy in ESCC cells. The expression of LC3 in the KYSE30 and KYSE270 cells exposed to echinatin was compared by immunofluorescence (**a**) and Western blot (**c**), **b** The number of LC3 puncta per cell and the percentage of the cells with LC3 puncta were counted under florescence microscope. KYSE30 and KYSE270 cells were treated with different concentrations of echinatin, with or without pretreated with BafA1 (0.5 nM, 12 h), and then the viability and colony-formatoin ability determined by CCK-8 assay (**d**) and colony-formation assay (**e**), respectively. Comparison of apoptosis and expression of apoptotic markers in ESCC cell treated with different concentrations of echinatin with or without BafA1 pretreatment (0.5 nM, 12 h) by an Annexin V-FITC/PI double staining assay (**f**) and western blot (**g**). Bar = 10 μm. Bars, SD; *P < 0.05, **P < 0. 01, ***P < 0.001.

We next examined whether echinatin could induce autophagy in ESCC cells. The confocal analysis showed that exposure of KYSE30 and KYSE270 cells to echinatin resulted in cellular LC3 puncta accumulation (Fig. 2a, b). In addition, the western blot results confirmed the increased LC3 expression in echinatin-treated cells in a dose-dependent manner (Fig. 2c). Furthermore, as shown in Fig. 2d, e, pretreatment with bafilomycinA1 (BafA1), an inhibitor of autophagy, could restore the cell viability and colony-formation ability in echinatin-treated KYSE30 and KYSE270 cells. The echinatin-induced apoptosis was reversed in presence of BafA1 treatment (Fig. 2f), and the echinatin-induced cleaved caspase-3 and cleaved PARP expressions were also significantly decreased after blockade of autophagy with BafA1 (Fig. 2g). These results indicate that echinatin induces ESCC cells apoptosis probably via the activation of autophagy.

### The AKT/mTOR signaling pathway mediates the effect of echinatin on autophagy and apoptosis in ESCC cells

**Fig. 3:**
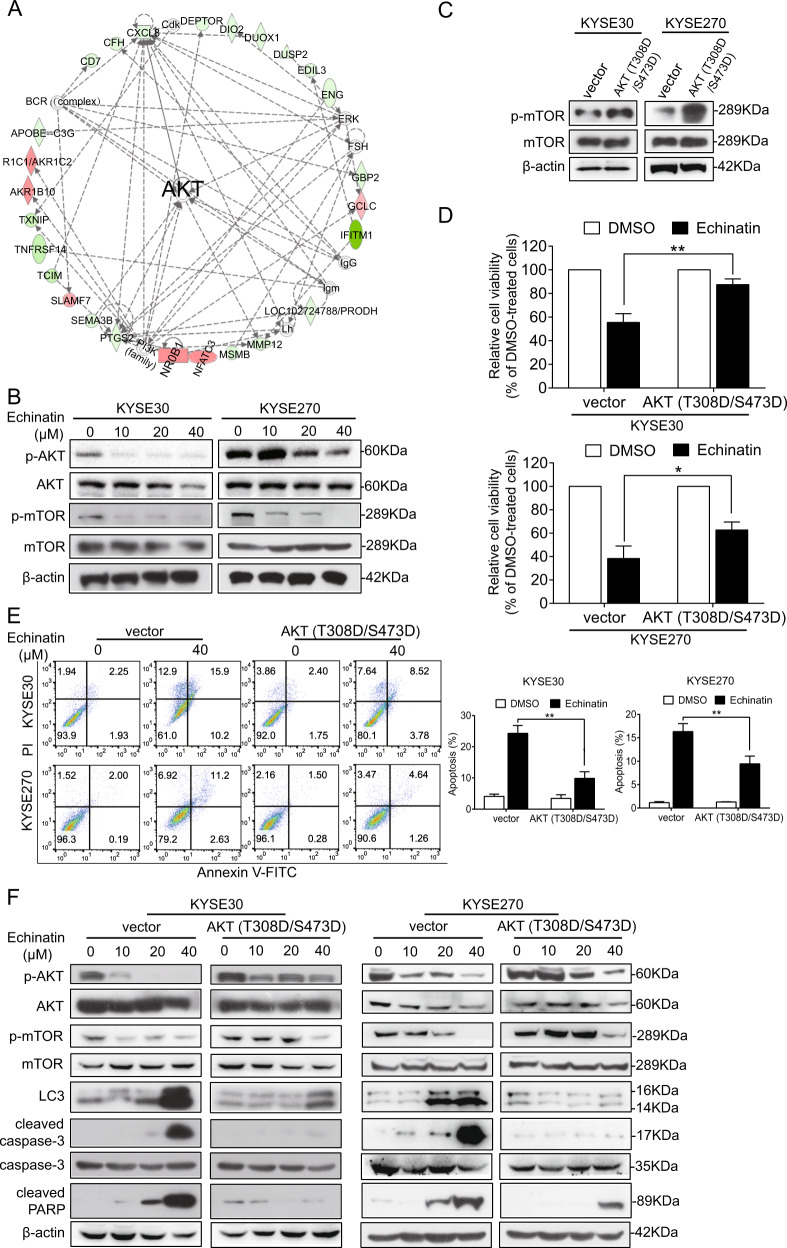
The AKT/mTOR signaling pathway mediates the effect of echinatin on autophagy and apoptosis in ESCC cells. **a** Ingenuity pathway analysis (IPA) suggested a dysregulation of AKT pathway in echinatin-treated KYSE30 cells. **b** KYSE30 and KYSE270 cells were exposed to different concentrations of echinatin for 48 h, and western blot analysis was performed to detect the expression levels of AKT, p-AKT, mTOR, and p-mTOR. **c–f** The KYSE30 and KYSE270 cells transfected with AKT (T308D/S473D)-expressing plasmid or vector control were treated with echinatin at the indicated concentrations for 48 h, and then compared for p-mTOR expression (**c**), cell viability (**d**), apoptosis (**e**), and expression levels of AKT, p-AKT, mTOR, p-mTOR, LC3, cleaved caspase-3 and cleaved PARP (**f**). Bars, SD; *P < 0.05, **P < 0. 01.

To investigate the molecular mechanisms of how echinatin induces apoptosis and autophagy in ESCC cells, the RNA-seq was performed to profile the differentially expressed genes in the KYSE30 cells treated with echinatin (20 μM) for 48 h. A total of 688 genes were identified to be significantly regulated by echinatin (fold change ≥4), including 105 upregulations and 583 downregulations (Supplementary Table S1). IPA analysis was used to characterize the canonical pathways. As shown in Fig. 3a, a cluster of echinatin-regulated genes constructed a signaling network that strongly pointed to the AKT pathway. As expected, echinatin was found to significantly reduce expression levels of p-AKT and p-mTOR (Fig. 3b), indicating that echinatin could inhibit mTOR/AKT pathway, which has been reported to inhibit autophagy in various types of cancer^23^. In order to investigate whether echinatin induces autophagy and apoptosis via AKT/mTOR pathway, a vector expressing AKT (T308D/S473D), the constitutively active form of AKT^24^, was transfected into KYSE30 and KYSE270 cells and the anticancer effects of echinatin were determined (Fig. 3c). The results showed that the ectopic expression of AKT (T308D/S473D) significantly increased the viability of echinatin-treated KYSE30 and KYSE270 cells (Fig. 3d). In addition, the echinatin-induced apoptosis was abrogated by AKT (T308D/S473D) overexpression (Fig. 3e), and the western blot assay further confirmed that echinatin-induced cleavage of caspase-3 and PARP were significantly decreased in AKT (T308D/S473D)-overexpressing ESCC cells (Fig. 3f). Moreover, increased LC3 protein expression upon echinatin treatment was also reversed by AKT (T308D/S473D) overexpression (Fig. 3f). Our results suggest that echinatin induces autophagic apoptosis in ESCC cells by inhibiting the AKT/mTOR pathway.

### Echinatin enhances the sensitivity of ESCC cells to fluorouracil (5-FU)

**Fig. 4:**
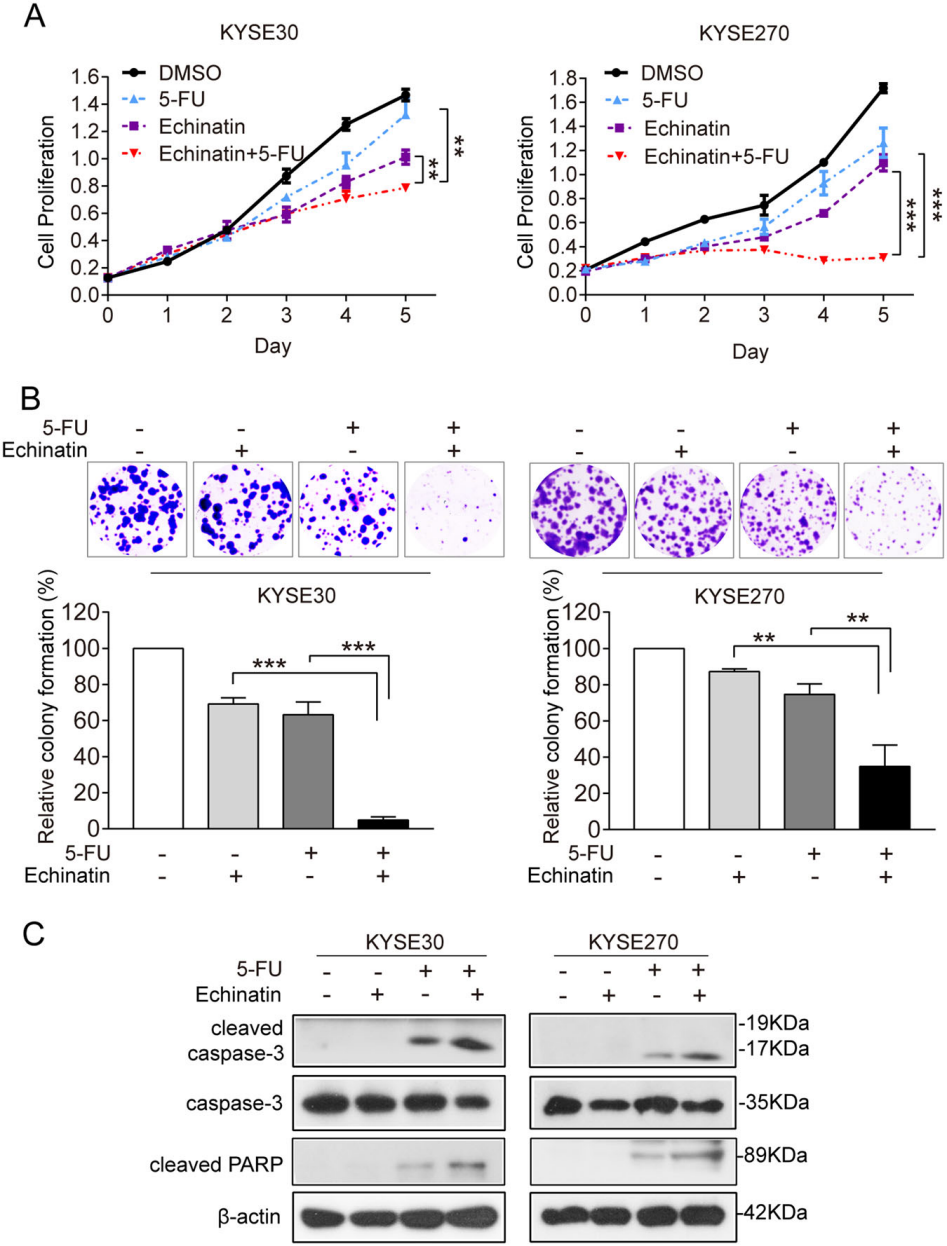
Echinatin enhances the sensitivity of ESCC cells to 5-FU. **a**, **b** The viability and colony-formation ability of the KYSE30 and KYSE270 cells treated with 5-FU (1.25 or 0.3 μM), echinatin (20 μM) alone, or the combination of 5-FU and echinatin for up to 5d was determined by CCK-8 assay (**a**) and colony-formation assay (**b**), respectively. **c** Echinatin increased apoptosis in the 5-FU-treated ESCC cells, indicated by higher expression levels of cleaved caspase-3 and cleaved PARP. Bars, SD; *P < 0.05, **P < 0. 01, ***P < 0.001.

The poor prognosis of ESCC was related to chemotherapy resistance. To study the biological function of echinatin in cancer chemoresistance, we examined the sensitivity of ESCC cells to 5-FU with or without echinatin by CCK-8, colony-formation and western blot assays. A combination of low-dose echinatin and low-dose 5-FU significantly suppressed cell growth (Fig. 4a) and colony formation (Fig. 4b) compared to low-dose echinatin or 5-FU alone. The expression of cleaved caspase-3 and cleaved PARP were increased in the ESCC cells treated with a combination of echinatin and 5-FU (Fig. 4c). Collectively, these data demonstrated that echinatin can increase the sensitivity of ESCC cells to 5-FU by inducing apoptosis.

### Echinatin inhibits migration and invasion in ESCC cells

**Fig. 5:**
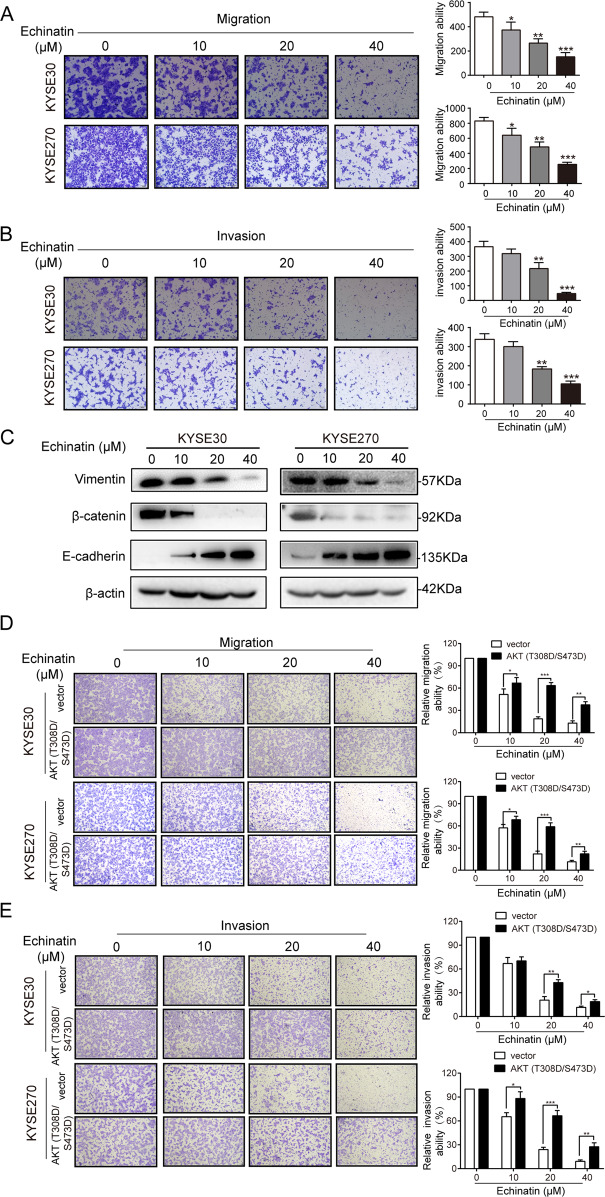
Echinatin inhibits migration and invasion in ESCC cells. The migration (**a**) and invasion (**b**) abilities of KYSE30 and KYSE270 cells treated with echinatin at different concentrations for 24 h were determined by chamber migration and invasion assays. **c** Western blot analysis of expressions of vimentin, β-catenin and E-cadherin in the KYSE30 and KYSE270 cells treated with echinatin at the indicated concentrations for 24 h. **d**, **e** The KYSE30 and KYSE270 cells transfected with AKT (T308D/S473D)-expressing plasmid or vector control were treated with echinatin at the indicated concentrations for 24 h, and then compared for migration ability (**d**), invasion ability (**e**). Bars, SD; *P < 0.05, **P < 0. 01, ***P < 0.001.

We next determined the effect of echinatin on the migration and invasion properties of ESCC cells, and the results showed that echinatin significantly inhibited the capability of KYSE30 and KYSE270 cells to migrate and invade (Fig. 5a, b). The expression of EMT markers, vimentin, β-catenin and E-cadherin, were also detected by western blot. The increased E-cadherin and the decreased β-catenin and vimentin expression indicated that echinatin inhibited the migration and invasion in ESCC cells by reversing EMT. In order to investigate whether echinatin inhibits migration and invasion via AKT pathway, the plasmid expression constitutively active form of AKT, AKT (T308D/S473D), was transfected into KYSE30 and KYSE270 cells, the results showed that activation of AKT signaling significantly abrogated the inhibitory effects of echinatin on ESCC cell migration and invasion (Fig. 5d, e).

### Echinatin suppresses growth of ESCC tumor xenografts in vivo

**Fig. 6:**
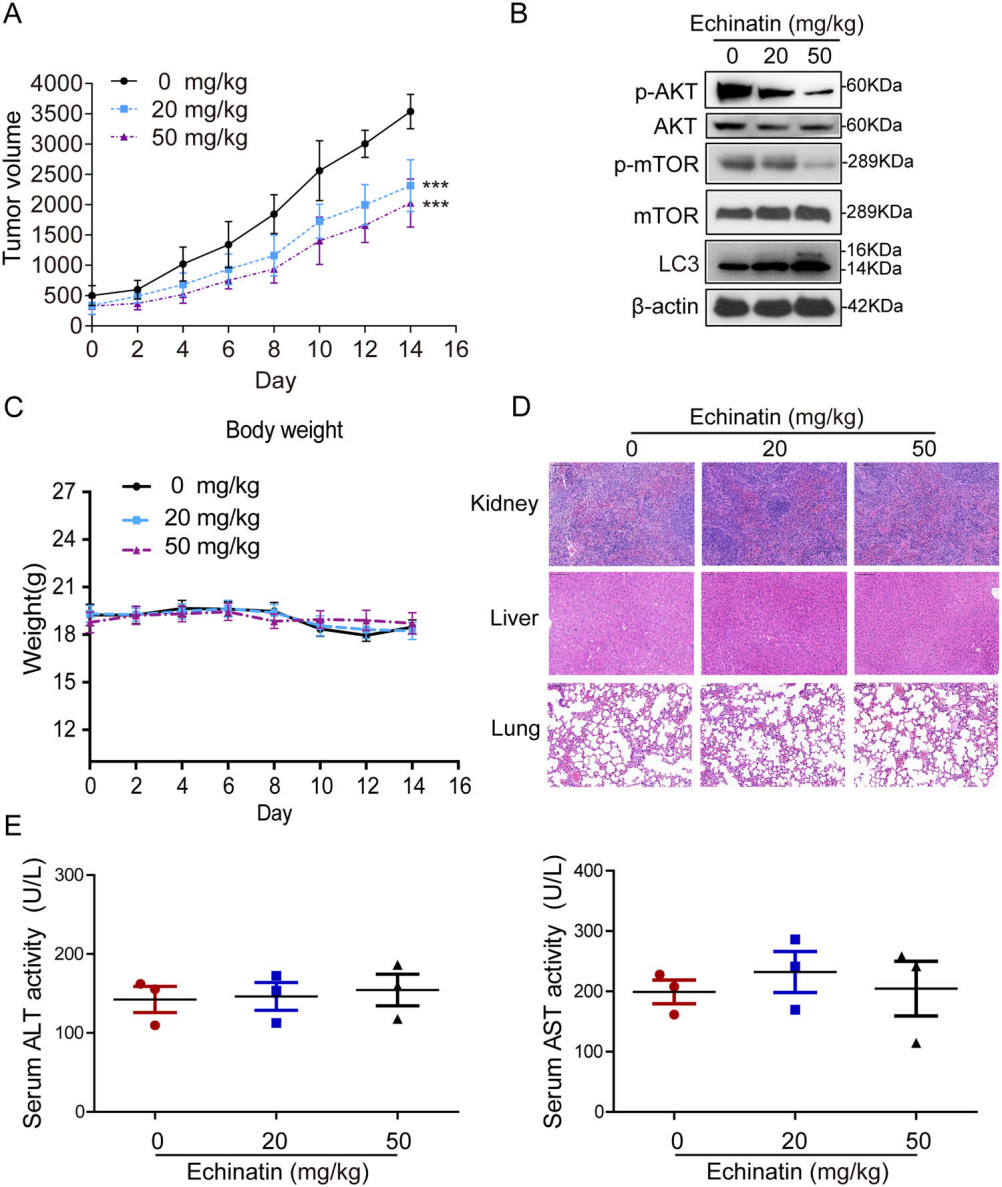
Echinatin suppresses the growth of ESCC tumor xenograft in nude mice. The nude mice bearing KYSE270-derived tumor xenografts were orally administrated with echinatin (20 mg/kg or 50 mg/kg) every 2 days (*n* = 6 per group), whereas the control group received the vehicle only. **a** Tumor curves showed that echinatin significantly suppressed the growth of tumor xenografts. **b** Expression levels of AKT, p-AKT, mTOR, p-mTOR and LC3 in the tumors from mice treated with echinatin or vehicle were detected by Western blot. **c** Body weight of nude mice during the experimental period. **d** Hematoxylin and eosin (H&E) staining of lung, liver, and kidney specimens collected from mice of the treatment and control groups. **e** Comparison of serum ALT and AST level between echinatin-treated and control groups. Bars, SD; ***P < 0.001.

The nude mice bearing KYSE270-derived tumor xenografts were orally administrated with echinatin (20 mg/kg and 50 mg/kg), and its effect on tumor growth was monitored. The tumor burden was found to be markedly suppressed with decreases of 57% and 48% in the group receiving 20 and 50 mg/kg of echinatin, respectively (Fig. 6a). Western blot data showed that echinatin inhibited the AKT/mTOR pathway, indicated by decreased expression levels of p-AKT and p-mTOR (Fig. 6b). There was no significant difference between the treatment and control groups in terms of body weight (Fig. 6c). Moreover, echinatin treatment did not exert any overt change in terms of the morphology of the vital organs, including lungs, liver, and kidneys (Fig. 6d). Furthermore, no significant difference in serum alanine transaminase (ALT) or aspartate transaminase (AST) levels of nude mice was observed (Fig. 6e), suggesting that echinatin had no toxic effect on animals.

## Discussion

Autophagy is a tightly regulated catabolic process of cellular self-digestion, in which cytoplasm, organelles, and proteins were separated into autophagosomes and then fused with lysosomes for degeneration to maintain cell renewal and homeostasis during starvation, stress, microbial invasion, and inflammation^25^. The dysregulation of autophagy is closely related to a number of diseases, including cancer, cardiovascular disease, and autoimmunity^26,27^. Many of the stimulators that lead to apoptosis can trigger autophagy, in which autophagy usually appeares before cell apoptosis. Autophagy activation beyond a certain threshold may lead to the breakdown of its functions, resulting in autophagic cell death or other types of cell death. The genetic experiments-related study also reported that autophagy may have an important role in controlling apoptosis^28^. Therefore, it has been well appreciated that autophagy can induce cell death to inhibit cancer progression, thus may be a target for cancer therapy^29,30^. In the present study, we found that echinatin increased the level of LC3, a marker protein of autophagy, indicating that echinatin can activate autophagy. In addition, when ESCC cells were pretreated with BafA1, an inhibitor of autophagy, the effects of echinatin on apoptosis, cell viability and colony-formation ability were markedly attenuated, indicating that echinatin acts as an activator of autophagy to induce apoptosis in ESCC cells.

Emerging evidences suggest that the AKT/mTOR pathway can activate a series of processes involved in tumor initiation and progression, including cell proliferation, differentiation, survival, chemoresistance and metastasis, and therefore may be a potential target for anticancer therapy^16,31,32^. To elucidate the action mechanism of echinatin in cancer cells, RNA-seq was performed to screen the differentially expressed genes in echinatin-treated ESCC cells, and IPA analysis suggested that AKT pathway, which has been documented to block autophagy through regulation of mTOR signaling^33^, may play an important role in the bioactivity of echinatin. The rescue experiment by using the AKT (T308D/S473D) plasmid expressing constitutively activation of AKT and the subsequent functional assays indicated that reactivation of AKT/mTOR signaling significantly abrogated the effects of echinatin on apoptosis, proliferation, autophagy, migration, and invasion abilities of cancer cells (Figs. 3, 5), suggesting that echinatin exerts anticancer effects through AKT/mTOR-autophagy pathway. The link between inflammation and cancer has been clearly demonstrated by epidemiological and experimental data^34,35^, for example, inflammatory bowel diseases such as Crohn’s disease and ulcerative colitis are associated with an increased risk of colon adenocarcinoma^36^. Some anti-inflammatory therapies showed great efficacy in cancer prevention and treatment^37^, which increases the rationale of study in anticancer bioactivity of anti-inflammatory drugs. The fact that echinatin was originally described as licorice extract that displayed antioxidant and anti-inflammatory activities, corroborates our finding on is anticancer effect. Furthermore, whether anticancer bioactivity of echinatin is related to its antioxidant and anti-inflammatory properties warrants further investigation.

Natural product such as traditional Chinese medicines has been used in the treatment of cancer for a long time. In view of its multi-component and multi-target characteristics in pharmacology, natural product is a promising resource for screening anticancer drugs. Vincristine, irinotecan, etoposide, and paclitaxel are classical examples of plant-derived compounds, and more new-generation drugs have been developed and used in clinic^11^. Echinatin is an active component of licorice, which possesses multiple biological activities. Although the chemistry of licorice has been extensively studied, the active components that contribute to its biological functions are far from clear. Licochalcone A, another flavonoid isolated from licorice, has been reported to inhibit cell viability in non-small cell lung cancer and breast cancer^21,38^. Although echinatin and licochalcone A have similar structures^39^, the anticancer activity of echinatin has not been reported up to now^12^. In this study, our results showed that echinatin decreased cell proliferation and colony formation in a dose-dependent manner, and more importantly, inhibited the growth of ESCC tumor xenografts in vivo, without observed damage to the normal esophageal epithelial cells or the vital organs of animals. Cancer, which still has no cure, has an increasing mortality rate year by year. Cancer metastasis and the resistance to chemotherapy often predict a poor prognosis^40^. Our data showed that echinatin not only significantly reduced the migration and invasion of ESCC cells, accompanied by EMT reversal, but also enhanced the antitumor effect of 5-FU, therefore, supporting the significance of echinatin in postoperative adjuvant therapy. Whether echinatin inhibits ESCC metastasis and sensitizes ESCC cells to 5-FU in vivo or not warrants further investigation.

**Fig. 7:**
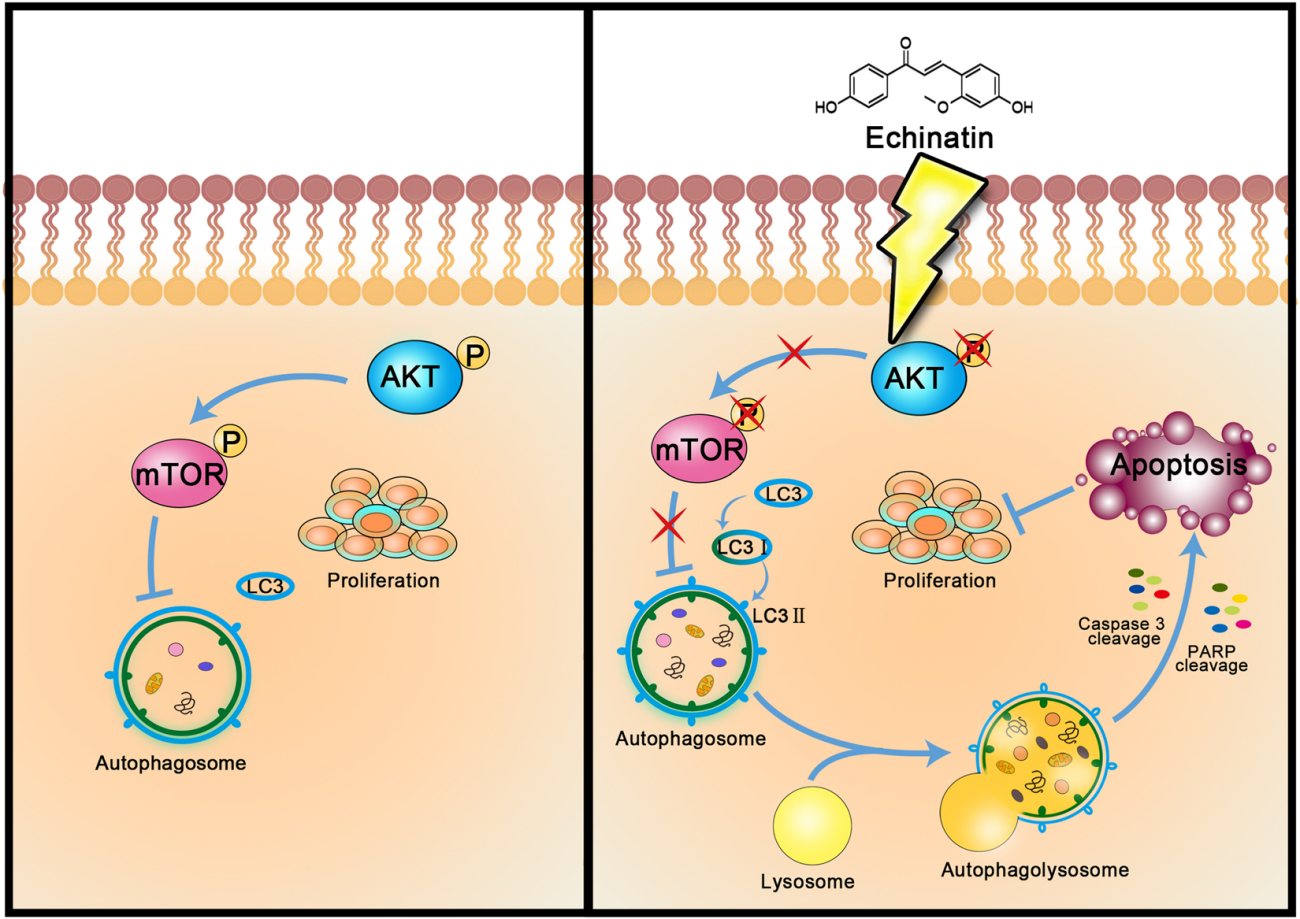
Schematic diagram summarizing the action mechanism of echinatin in cancer cells. Activation of AKT/mTOR signaling inhibits autophagy to regulate the balance between proliferation and apoptosis in ESCC cells, while echinatin induces cell autophagy and apoptosis via inhibiting AKT/mTOR pathway, leading to the suppression of cell growth and tumorigensis.

Taken together, we uncovered that echinatin is a potential anticancer natural product with an inhibitory effect on ESCC tumorigenesis and metastatic potential, and sensitizes ESCC cells to 5-FU treatment. Mechanistically, echinatin induces cell autophagy and apoptosis via the AKT/mTOR signaling pathway (Fig. 7). It is the first time to demonstrate the anticancer bioactivity of echinatin and the underlying mechanism. These findings provide solid evidence on the potential of echinatin as therapeutic options for the treatment of ESCC.

## Materials and methods

### Cell culture and treatment

Human ESCC cell lines, KYSE30 and KYSE270 were obtained from DSMZ (Braunschweig, Germany). The cell lines were authenticated by short tandem repeat profiling, and tested for mycoplasma contamination. These cells were cultured in RPMI 1640 medium supplemented with 10% fetal bovine serum (Thermo Fisher Scientific, Waltham, MA, USA) and penicillin and streptomycin at 37 °C in 5% CO_2_.

### Plasmid and transfection

The plasmid expressing constitutively active form of AKT, the AKT (T308D/S473D), was a gift from Dr. Robert Weinberg^41^. ESCC cells were transfected with AKT (T308D/S473D) or vector control using Lipofectamine 3000 reagent (Invitrogen, Carlsbad, CA, USA) according to the manufacturer’s instructions.

### Cell viability assay

Cells were cultured in 96-well plate for 24 h, and then treated with echinatin (TargetMol, Boston, MA, USA) at different concentrations. The cell viability was determined by Cell Counting Kit-8 (CCK-8; Dojindo Molecular Technologies Inc., Rockville, MD, USA) according to the manufacturer’s guidelines, and the absorbance was quantified by an automated microplate spectrophotometer (BioTek Instruments Winooski, VT, USA) at 450 nm^22^.

### Colony-formation assay

Colony-formation assay was performed as described previously^4^. Cells were seeded in six-cell plate and cultured with echinatin. After 14 days, the cells were washed gently with PBS three times, fixed with 75% ethanol for 15 min, and stained with crystal violet. The numbers of colonies were counted for analysis.

### Annexin V-FITC/PI staining assay

Cell apoptosis was determined using an Annexin V-FITC/PI Apoptosis Detection kit (KeyGen, Nanjing, Jiangsu, China)^4^. In brief, cells were suspended in binding buffer and stained with annexin V-FITC and propidium iodide (PI) for 15 min at room temperature in darkness. Apoptosis was assessed and analyzed using a C6 flow cytometer (BD Biosciences, San Diego, CA, USA).

### Immunofluorescence staining

The cells were fixed with 4% paraformaldehyde for 10 min, and blocked with 10% goat serum for 2 h after being permeabilized with 0.1% TrintonX-100 for 10 min. After washed with 1% TBST three times, cells were incubated with the primary antibody against LC3A/B at 4 °C overnight, and then incubated with the appropriate fluorescent secondary antibody at room temperature for 2 h. The cells were counter-stained with 40,6-diamidino-2-phenylindole (DAPI, Thermo Fisher Scientific, Waltham, MA, USA), and the images were visualized by laser scanning confocal microscopy (Carl Zeiss AG, Jena, Thuringia, Germany)^26^.

### RNA-seq and IPA

The RNA-seq was performed in the Beijing Genomics Institute Tech (Shenzhen, Guangzhou, China). Gene profiles in two conditions were analyzed by DESeq R package (1.10.1). The genes with a *P* value less than 0.05 determined by DESeq were represented as differentially expressed. The IPA software (Ingenuity Systems, Redwood City, CA, USA) was used for pathway analysis^16^.

### The detection of cell migration and invasion assay in vitro

Cell migration ability was detected by using uncoated Transwell chambers (8 μm pore size; BD Biosciences, Bedford, MA, USA). Cells in serum-free medium were seeded in the upper chamber, and the complete growth medium as the chemoattractant was added to the lower chamber. After 24 h, the migrated cells were fixed with methanol and then stained with crystal violet. The images of each well were obtained in three different fields. The matrigel-coated chambers were used in invasion assay with a similar protocol in accordance with cell migration assay^42^.

### Western blot analysis

Preparation of cell lysates and details of western blot were described previously^43^. The antibodies used included E-cadherin (1:5000; Proteintech, 20874-1-AP), β-Catenin (1:1000, Cell Signaling Technology, CST9562), vimentin (1:1000, Cell Signaling Technology, CST5741), LC3A/B (1:1000, Cell Signaling Technology, CST4108), caspase-3 (1:1000, Cell Signaling Technology, CST9662), cleaved caspase-3 (1:1000, Cell Signaling Technology, CST 9661), PARP (1:1000, Cell Signaling Technology, CST9532), cleaved PARP (1:1000, Cell Signaling Technology, CST 5625), phospho-AKT (p-AKT) (1:2000, Cell Signaling Technology, CST4060), phospho-mTOR (p-mTOR) (1:1000, Cell Signaling Technology, CST5536), mTOR (1:1000, Cell Signaling Technology, CST2972) and AKT (1:2000, Cell Signaling Technology, CST2920). The reaction was visualized using Clarity Western ECL substrate (Bio-Rad, Hercules, CA, USA) and detected by exposure to autoradiographic film.

### Tumor xenograft experiment

Tumor xenograft experiments were performed as described previously^44,45^. The male nude mice aged 6–8 weeks were maintained in standard conditions and cared for according to the institutional guidelines for animal care. KYSE270 cells in PBS and matrigel with equal volume were subcutaneously injected into mice to establish tumor xenografts. When the diameter of the tumor reached about 5 mm, the mice were randomly divided into three groups (*n* = 6 mice/group) and treated with echinatin (20 mg/kg and 50 mg/kg) by oral gavage, as well as vehicle as control, respectively, every 2 days. Tumor volumes were measured every 2 days in a blinded fashion, calculated by the formula of *V* = (length × width^2^)/2. At the end of the experiment, the tumors, livers, lungs, and kidneys were collected for western blot and histological analysis. All the animal experiments were approved by the Ethics Committee for Animal Experiments of Jinan University.

### Statistical analysis

All in vitro experiments were performed in three independent experiments. Data were expressed as the mean ± SD and compared by GraphPad Prism 7.0 (GraphPad Software Inc., San Diego, CA, USA). Comparison between groups were analyzed by a Student’s *t* test method. *P* values < 0.05 were deemed significant.

## Supplementary information

Supplementary Figure legend

Supplementary Figure S1

Supplementary Table S1
