# Supplementary material for: Echinatin suppresses esophageal cancer tumor growth and invasion through inducing AKT/mTOR-dependent autophagy and apoptosis
Source: Cell Death Dis. 2020 Jul 13;11(7):524. doi: 10.1038/s41419-020-2730-7 (PMC7354992; doi:10.1038/s41419-020-2730-7)
Supplement: Supplementary file 1 — Supplementary Figure legend [file 41419_2020_2730_MOESM1_ESM.docx]

**Figure S1.** (A) The detection of apoptosis in ESCC cells treated with different concentrations of echinatin for 24 h by an Annexin V-FITC/PI double staining assay. (B) Western blot analysis was performed to detect the expression of cleaved caspase-3, caspase-3 and cleaved PARP in the ESCC cells treated with indicated concentrations of echinatin for 24 h. Bars, SD; ** P < 0. 01, ***, P < 0.001.
