# Supplementary figures and images for: Echinatin suppresses esophageal cancer tumor growth and invasion through inducing AKT/mTOR-dependent autophagy and apoptosis
Source: Cell Death Dis. 2020 Jul 13;11(7):524. doi: 10.1038/s41419-020-2730-7 (PMC7354992; doi:10.1038/s41419-020-2730-7)

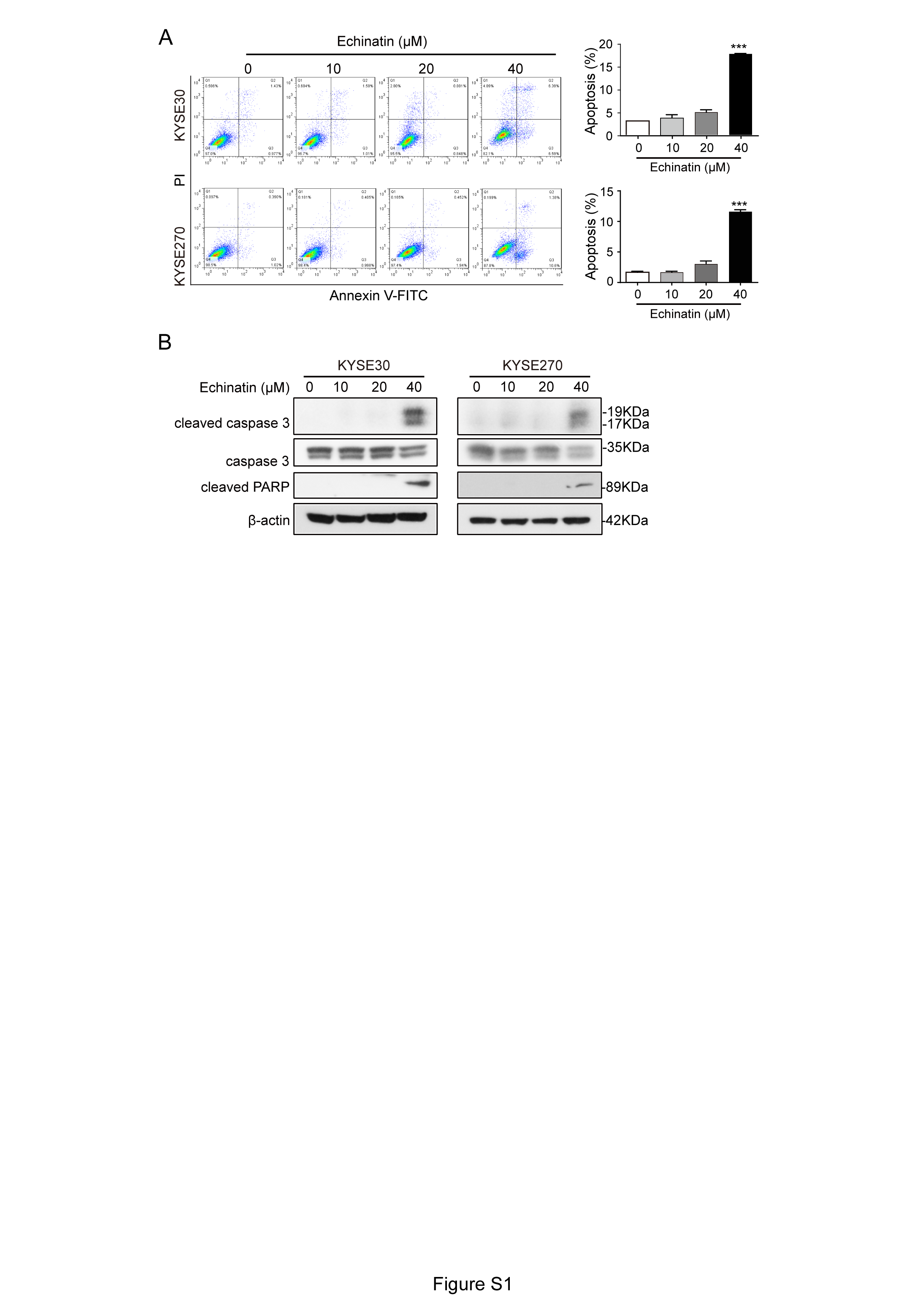

Supplement: Supplementary file 2 — Supplementary Figure S1 [file 41419_2020_2730_MOESM2_ESM.tif]
