# Supplementary material for: Echinatin suppresses esophageal cancer tumor growth and invasion through inducing AKT/mTOR-dependent autophagy and apoptosis
Source: Cell Death Dis. 2020 Jul 13;11(7):524. doi: 10.1038/s41419-020-2730-7 (PMC7354992; doi:10.1038/s41419-020-2730-7)
Supplement: Supplementary file 3 — Supplementary Table S1 [file 41419_2020_2730_MOESM3_ESM.docx]

**Supplementary Table S1.** Differentially expressed genes (fold change ≥ 4.0) in the KYSE30 cells treated with echinatin (20 μM) for 48 h.

| **ID** | **Symbol** | **P value** | **Fold Change** |
| --- | --- | --- | --- |
| 80201 | HKDC1 | 8.06E-47 | 41.6666667 |
| 94031 | HTRA3 | 3.63E-05 | 19 |
| BGI_novel_G000130 | BGI_novel_G000130 | 3.53E-11 | 18.3333333 |
| BGI_novel_G001214 | BGI_novel_G001214 | 1.24E-33 | 18.2666667 |
| BGI_novel_G000493 | BGI_novel_G000493 | 6.60E-206 | 16.7272727 |
| 100873985 | MED14OS | 0.00011773 | 16.3333333 |
| 3162 | HMOX1 | 0 | 15.8213689 |
| 114928 | GPRASP2 | 6.66E-33 | 15.3333333 |
| 11000 | SLC27A3 | 2.93E-87 | 13.7666667 |
| 10485 | C1orf61 | 7.36E-08 | 13.625 |
| 128312 | HIST3H2BB | 0.00213312 | 11.4285714 |
| 29948 | OSGIN1 | 0 | 10.9058172 |
| 8370 | HIST2H4A | 7.41E-07 | 10.7142857 |
| 554313 | HIST2H4B | 7.41E-07 | 10.7142857 |
| BGI_novel_G000013 | BGI_novel_G000013 | 1.48E-07 | 10.7142857 |
| BGI_novel_G001238 | BGI_novel_G001238 | 3.23E-19 | 10.5762712 |
| 408050 | NOMO3 | 3.98E-09 | 10.5 |
| 190 | NR0B1 | 0.00035684 | 9.33333333 |
| BGI_novel_G000573 | BGI_novel_G000573 | 4.43E-49 | 9.0625 |
| 1645 | AKR1C1 | 7.37E-31 | 8.65714286 |
| 57016 | AKR1B10 | 7.97E-35 | 8.57142857 |
| 375033 | PEAR1 | 7.37E-07 | 8.5 |
| 392617 | ELFN1 | 0.0086479 | 8 |
| 23208 | SYT11 | 0.0022458 | 8 |
| 342931 | RFPL4A | 0.0202092 | 8 |
| 1646 | AKR1C2 | 9.65E-34 | 7.83333333 |
| 353137 | LCE1F | 0.0202092 | 7.8 |
| 4051 | CYP4F3 | 8.56E-125 | 7.16842105 |
| 1543 | CYP1A1 | 4.19E-89 | 7.08450704 |
| BGI_novel_G000524 | BGI_novel_G000524 | 0.0599044 | 7 |
| 57823 | SLAMF7 | 0.0599044 | 7 |
| 7047 | TGM4 | 0.0116112 | 7 |
| 100653515 | CEP295NL | 0.1017186 | 7 |
| 56300 | IL36G | 0.0599044 | 7 |
| 8644 | AKR1C3 | 3.78E-07 | 7 |
| 54550 | NECAB2 | 3.87E-18 | 7 |
| 9363 | RAB33A | 0.00017355 | 7 |
| BGI_novel_G001191 | BGI_novel_G001191 | 1.87E-118 | 6.50564334 |
| 100302736 | TMED7-TICAM2 | 3.33E-120 | 6.2 |
| 3231 | HOXD1 | 0.1706486 | 6 |
| 26095 | PTPN20 | 0.1706486 | 6 |
| 60401 | EDA2R | 0.038994 | 6 |
| 85366 | MYLK2 | 0.00305288 | 6 |
| 2878 | GPX3 | 0.1706486 | 6 |
| 3004 | GZMM | 0.0599044 | 6 |
| 57834 | CYP4F11 | 0 | 5.75129534 |
| 25858 | TEX40 | 0.0599044 | 5.75 |
| 100287036 | LOC100287036 | 0.00081847 | 5.71428571 |
| BGI_novel_G001220 | BGI_novel_G001220 | 1.35E-40 | 5.67307692 |
| 2729 | GCLC | 0 | 5.6231386 |
| BGI_novel_G000694 | BGI_novel_G000694 | 1.04E-05 | 5.31034483 |
| BGI_novel_G001192 | BGI_novel_G001192 | 0.0599044 | 5.25 |
| 140809 | SRXN1 | 0 | 5.21906005 |
| 147166 | TRIM16L | 0 | 5.21738333 |
| BGI_novel_G001239 | BGI_novel_G001239 | 3.36E-08 | 5.12820513 |
| 8808 | IL1RL2 | 0.28196 | 5 |
| 2788 | GNG7 | 0.038994 | 5 |
| 100534592 | URGCP-MRPS24 | 5.48E-08 | 5 |
| 55301 | OLAH | 0.1017186 | 5 |
| 6869 | TACR1 | 0.038994 | 5 |
| BGI_novel_G001044 | BGI_novel_G001044 | 0.0249508 | 5 |
| 145226 | RDH12 | 0.28196 | 5 |
| 6580 | SLC22A1 | 0.28196 | 5 |
| 117248 | GALNT15 | 0.00668892 | 5 |
| 2918 | GRM8 | 0.0349354 | 5 |
| 4759 | NEU2 | 0.1706486 | 5 |
| 80045 | GPR157 | 2.03E-15 | 4.97435897 |
| 4775 | NFATC3 | 1.01E-239 | 4.97222222 |
| 8681 | JMJD7-PLA2G4B | 5.32E-34 | 4.94594595 |
| 2730 | GCLM | 0 | 4.86666667 |
| 100526832 | PHOSPHO2-KLHL23 | 2.20E-47 | 4.72340426 |
| 57057 | TBX20 | 9.72E-07 | 4.69230769 |
| 22979 | EFR3B | 7.83E-17 | 4.66666667 |
| 8878 | SQSTM1 | 0 | 4.58565095 |
| 644054 | FAM25C | 0.1706486 | 4.55555556 |
| BGI_novel_G000645 | BGI_novel_G000645 | 0.1706486 | 4.5 |
| 347517 | RAB41 | 0.1017186 | 4.33333333 |
| 390010 | NKX1-2 | 0.1706486 | 4.33333333 |
| BGI_novel_G000185 | BGI_novel_G000185 | 0.0349354 | 4.25 |
| 55068 | ENOX1 | 0.00011559 | 4.16666667 |
| 84220 | RGPD5 | 1.22E-79 | 4.08474576 |
| 8970 | HIST1H2BJ | 2.06E-13 | 4.07142857 |
| BGI_novel_G001277 | BGI_novel_G001277 | 6.82E-06 | 4.03333333 |
| 57167 | SALL4 | 0.1012024 | 4 |
| 653140 | FAM228A | 0.456714 | 4 |
| 374786 | EFCAB5 | 0.1012024 | 4 |
| 8329 | HIST1H2AI | 5.56E-05 | 4 |
| 56253 | CRTAM | 0.456714 | 4 |
| 257062 | CATSPERD | 0.28196 | 4 |
| 10324 | KLHL41 | 0.0249508 | 4 |
| 9290 | GPR55 | 0.248068 | 4 |
| 51237 | MZB1 | 0.28196 | 4 |
| 387755 | INSC | 0.00254148 | 4 |
| 9389 | SLC22A14 | 0.28196 | 4 |
| 442247 | RFPL4B | 0.456714 | 4 |
| 80763 | SPX | 0.1012024 | 4 |
| 79442 | LRRC2 | 0.038994 | 4 |
| 138240 | C9orf57 | 0.1706486 | 4 |
| 84436 | ZNF528 | 0.0599044 | 4 |
| 170690 | ADAMTS16 | 0.0349354 | 4 |
| 25900 | IFFO1 | 0.1706486 | 4 |
| BGI_novel_G000623 | BGI_novel_G000623 | 0.30275 | -4 |
| 10636 | RGS14 | 0.1661948 | -4 |
| 100506115 | LINC01272 | 0.126212 | -4 |
| 2912 | GRM2 | 0.1661948 | -4 |
| 140876 | FAM65C | 0.126212 | -4 |
| 1844 | DUSP2 | 5.55E-36 | -4 |
| 9120 | SLC16A6 | 4.48E-07 | -4 |
| 387885 | CFAP73 | 0.1661948 | -4 |
| 2563 | GABRD | 0.00527042 | -4 |
| 11202 | KLK8 | 0.00171138 | -4 |
| 255239 | ANKK1 | 0.1661948 | -4 |
| BGI_novel_G001452 | BGI_novel_G001452 | 0.126212 | -4 |
| 439996 | IFIT1B | 0.126212 | -4 |
| 29933 | GPR132 | 0.126212 | -4 |
| 9900 | SV2A | 0.156618 | -4 |
| 53905 | DUOX1 | 0.0950816 | -4 |
| 347252 | IGFBPL1 | 0.215574 | -4 |
| 222662 | LHFPL5 | 0.0723032 | -4 |
| 125981 | ACER1 | 0.30275 | -4 |
| BGI_novel_G001436 | BGI_novel_G001436 | 1.09E-06 | -4 |
| 825 | CAPN3 | 0.30275 | -4 |
| 55515 | ASIC4 | 0.01557568 | -4 |
| 6641 | SNTB1 | 9.29E-05 | -4 |
| 8477 | GPR65 | 0.0246492 | -4 |
| 56171 | DNAH7 | 2.69E-05 | -4 |
| 3745 | KCNB1 | 0.00126988 | -4 |
| BGI_novel_G000536 | BGI_novel_G000536 | 0.206596 | -4 |
| 9843 | HEPH | 0.0723032 | -4 |
| 4843 | NOS2 | 0.0472188 | -4 |
| 1828 | DSG1 | 0.00380494 | -4 |
| 221400 | TDRD6 | 0.01248036 | -4 |
| 64600 | PLA2G2F | 0.1661948 | -4 |
| 51411 | BIN2 | 0.00215188 | -4 |
| 2977 | GUCY1A2 | 8.06E-06 | -4 |
| 146853 | C17orf50 | 0.30275 | -4 |
| BGI_novel_G000280 | BGI_novel_G000280 | 0.126212 | -4 |
| 22987 | SV2C | 0.00187457 | -4 |
| 100131539 | ZNF705E | 0.00745744 | -4 |
| 5697 | PYY | 0.01907858 | -4 |
| BGI_novel_G001476 | BGI_novel_G001476 | 0.01085394 | -4 |
| 129025 | ZNF280A | 0.30275 | -4 |
| 283748 | PLA2G4D | 0.126212 | -4 |
| 3569 | IL6 | 0.0723032 | -4 |
| 56123 | PCDHB13 | 0.01907858 | -4 |
| 56133 | PCDHB2 | 0.022553 | -4 |
| 9496 | TBX4 | 0.0893074 | -4 |
| 84125 | LRRIQ1 | 4.45E-08 | -4 |
| 939 | CD27 | 0.1661948 | -4 |
| 27185 | DISC1 | 0.033674 | -4 |
| 161497 | STRC | 0.056584 | -4 |
| 57571 | CARNS1 | 0.0262902 | -4 |
| 6564 | SLC15A1 | 0.215574 | -4 |
| BGI_novel_G000106 | BGI_novel_G000106 | 0.0246492 | -4 |
| 51207 | DUSP13 | 0.0331016 | -4 |
| 83450 | DRC3 | 4.16E-12 | -4 |
| 84073 | MYCBPAP | 0.00215188 | -4 |
| 123 | PLIN2 | 0.30275 | -4 |
| 5342 | PLGLB2 | 3.24E-05 | -4 |
| 10656 | KHDRBS3 | 0.30275 | -4 |
| 57119 | EPPIN | 0.535578 | -4 |
| 6954 | TCP11 | 0.30275 | -4 |
| 63895 | PIEZO2 | 0.613834 | -4 |
| 4065 | LY75 | 0.0331016 | -4 |
| 84639 | IL1F10 | 0.30275 | -4 |
| 11148 | HHLA2 | 0.00257888 | -4 |
| 27189 | IL17C | 0.30275 | -4 |
| 57007 | ACKR3 | 0.30275 | -4 |
| 27319 | BHLHE22 | 0.215574 | -4 |
| 84229 | DRC7 | 0.1661948 | -4 |
| 728927 | ZNF736 | 0.01609902 | -4 |
| 266722 | HS6ST3 | 0.0262902 | -4 |
| 7780 | SLC30A2 | 0.0723032 | -4 |
| 340273 | ABCB5 | 0.00149511 | -4 |
| 2201 | FBN2 | 0.00745744 | -4 |
| 56114 | PCDHGA1 | 0.0723032 | -4 |
| 91807 | MYLK3 | 0.01907858 | -4 |
| 229 | ALDOB | 0.1661948 | -4 |
| 146225 | CMTM2 | 0.1661948 | -4 |
| 129807 | NEU4 | 0.126212 | -4 |
| 353189 | SLCO4C1 | 0.0406852 | -4 |
| 1384 | CRAT | 6.35E-11 | -4 |
| 24 | ABCA4 | 4.67E-05 | -4 |
| 1259 | CNGA1 | 1.32E-12 | -4.0126582 |
| 440585 | FAM183A | 1.64E-05 | -4.025641 |
| 3576 | CXCL8 | 3.80E-99 | -4.0402477 |
| 2335 | FN1 | 2.54E-148 | -4.0416667 |
| 8309 | ACOX2 | 1.45E-15 | -4.0606061 |
| 7447 | VSNL1 | 3.89E-07 | -4.0625 |
| BGI_novel_G000263 | BGI_novel_G000263 | 1.48E-22 | -4.0718563 |
| 12 | SERPINA3 | 6.99E-08 | -4.0909091 |
| 8356 | HIST1H3J | 0.0723032 | -4.0909091 |
| 5521 | PPP2R2B | 1.64E-05 | -4.0909091 |
| BGI_novel_G000555 | BGI_novel_G000555 | 1.98E-09 | -4.1 |
| 5625 | PRODH | 1.23E-11 | -4.1 |
| 5003 | SLC22A18AS | 0.00025999 | -4.1111111 |
| 201501 | ZBTB7C | 2.84E-07 | -4.125 |
| 60489 | APOBEC3G | 0.00043157 | -4.125 |
| 100505741 | SPATA1 | 5.50E-05 | -4.1428571 |
| 22977 | AKR7A3 | 0.00911198 | -4.1428571 |
| 7025 | NR2F1 | 2.12E-40 | -4.1454545 |
| 5743 | PTGS2 | 6.87E-72 | -4.1604938 |
| 55815 | TSNAXIP1 | 0.00043157 | -4.1666667 |
| 11173 | ADAMTS7 | 1.18E-07 | -4.1666667 |
| 221091 | LRRN4CL | 0.00014248 | -4.1666667 |
| 84293 | FAM213A | 5.71E-77 | -4.1814346 |
| 114769 | CARD16 | 0.00043157 | -4.1818182 |
| 2982 | GUCY1A3 | 2.71E-16 | -4.1818182 |
| 64108 | RTP4 | 1.25E-08 | -4.2105263 |
| 100132074 | FOXO6 | 1.85E-90 | -4.2285714 |
| 29943 | PADI1 | 1.18E-79 | -4.2340426 |
| 9635 | CLCA2 | 7.77E-70 | -4.2345679 |
| 28951 | TRIB2 | 1.22E-293 | -4.2359551 |
| 441054 | C4orf47 | 4.93E-06 | -4.24 |
| 51351 | ZNF117 | 8.21E-58 | -4.245283 |
| 10410 | IFITM3 | 0 | -4.2486943 |
| 165545 | DQX1 | 0.00440746 | -4.25 |
| 4837 | NNMT | 0.0331016 | -4.25 |
| 3883 | KRT33A | 0.0406852 | -4.25 |
| 56898 | BDH2 | 6.32E-48 | -4.2531646 |
| 2022 | ENG | 7.18E-113 | -4.2575758 |
| 257169 | C9orf43 | 3.15E-10 | -4.2727273 |
| 64798 | DEPTOR | 3.94E-38 | -4.2727273 |
| 3821 | KLRC1 | 5.11E-07 | -4.2857143 |
| 11023 | VAX1 | 0.00105817 | -4.2857143 |
| 80310 | PDGFD | 3.75E-25 | -4.3103448 |
| 8581 | LY6D | 0 | -4.3253355 |
| 2920 | CXCL2 | 0.01085394 | -4.3333333 |
| 10896 | OCLM | 0.1661948 | -4.3333333 |
| BGI_novel_G000559 | BGI_novel_G000559 | 0.0723032 | -4.3333333 |
| 222950 | NYAP1 | 0.00149511 | -4.3333333 |
| 127707 | KLHDC7A | 3.04E-08 | -4.3333333 |
| BGI_novel_G000240 | BGI_novel_G000240 | 0.01907858 | -4.3333333 |
| 8335 | HIST1H2AB | 0.1661948 | -4.3333333 |
| BGI_novel_G001153 | BGI_novel_G001153 | 0.0123438 | -4.3333333 |
| 3485 | IGFBP2 | 6.08E-193 | -4.3498965 |
| 342979 | PALM3 | 2.25E-09 | -4.3529412 |
| 90134 | KCNH7 | 3.35E-13 | -4.3529412 |
| 23037 | PDZD2 | 1.44E-29 | -4.3636364 |
| 1961 | EGR4 | 2.15E-05 | -4.375 |
| 8510 | MMP23B | 2.98E-07 | -4.3913043 |
| 10002 | NR2E3 | 0.00171138 | -4.4 |
| 441476 | STPG3 | 0.0123438 | -4.4 |
| 11283 | CYP4F8 | 0.0030173 | -4.4 |
| 115557 | ARHGEF25 | 0.00018529 | -4.4285714 |
| 5176 | SERPINF1 | 1.32E-37 | -4.4409449 |
| 23089 | PEG10 | 0 | -4.4428898 |
| 57523 | NYNRIN | 2.11E-110 | -4.4603175 |
| 59 | ACTA2 | 1.39E-18 | -4.4666667 |
| 25840 | METTL7A | 4.61E-294 | -4.4769231 |
| 375189 | PFN4 | 4.83E-09 | -4.4782609 |
| 128272 | ARHGEF19 | 2.85E-183 | -4.4837398 |
| 5348 | FXYD1 | 1.11E-06 | -4.5 |
| 1734 | DIO2 | 0.00126988 | -4.5 |
| 85446 | ZFHX2 | 4.94E-10 | -4.5 |
| 5333 | PLCD1 | 0.0123438 | -4.5 |
| 2042 | EPHA3 | 2.90E-06 | -4.5 |
| 83872 | HMCN1 | 2.27E-08 | -4.5 |
| BGI_novel_G000229 | BGI_novel_G000229 | 0.022553 | -4.5 |
| 4901 | NRL | 0.00339824 | -4.5 |
| 2568 | GABRP | 0.01907858 | -4.5 |
| 785 | CACNB4 | 6.06E-08 | -4.5 |
| BGI_novel_G000006 | BGI_novel_G000006 | 0.30275 | -4.5 |
| 1392 | CRH | 0.0893074 | -4.5 |
| 113730 | KLHDC7B | 0.0331016 | -4.5 |
| 3237 | HOXD11 | 0.00085947 | -4.5 |
| 943 | TNFRSF8 | 0.01557568 | -4.5 |
| 971 | CD72 | 0.01907858 | -4.5 |
| 590 | BCHE | 2.60E-10 | -4.5 |
| 116285 | ACSM1 | 0.00911198 | -4.5 |
| 4049 | LTA | 0.0893074 | -4.5 |
| 1776 | DNASE1L3 | 0.0723032 | -4.5 |
| 326624 | RAB37 | 0.022553 | -4.5 |
| 196996 | GRAMD2 | 0.00043157 | -4.5 |
| 2843 | GPR20 | 0.0893074 | -4.5 |
| BGI_novel_G000314 | BGI_novel_G000314 | 0.00187457 | -4.5 |
| 1512 | CTSH | 0.0030173 | -4.5 |
| 219621 | C10orf107 | 0.00610434 | -4.5 |
| 56477 | CCL28 | 0.00031248 | -4.5 |
| BGI_novel_G000809 | BGI_novel_G000809 | 4.63E-115 | -4.5137615 |
| 57468 | SLC12A5 | 3.37E-44 | -4.5339806 |
| 25928 | SOSTDC1 | 5.74E-17 | -4.5405405 |
| 3456 | IFNB1 | 1.00E-07 | -4.5428571 |
| 168544 | ZNF467 | 1.84E-56 | -4.5564516 |
| 401399 | PRRT4 | 3.83E-09 | -4.5714286 |
| 283212 | KLHL35 | 4.34E-12 | -4.5833333 |
| 56146 | PCDHA2 | 5.50E-20 | -4.5833333 |
| 5972 | REN | 0.00610434 | -4.6 |
| 1410 | CRYAB | 0.01273574 | -4.6 |
| 7379 | UPK2 | 3.29E-77 | -4.6199461 |
| 3761 | KCNJ4 | 4.68E-08 | -4.625 |
| 6659 | SOX4 | 1.00E-270 | -4.6565217 |
| 56 | ACRV1 | 0.0893074 | -4.6666667 |
| 642778 | NPIPA3 | 0.0893074 | -4.6666667 |
| 718 | C3 | 1.28E-16 | -4.6666667 |
| BGI_novel_G000626 | BGI_novel_G000626 | 0.1661948 | -4.6666667 |
| 2020 | EN2 | 0.00149511 | -4.6666667 |
| 130752 | MDH1B | 0.00339824 | -4.6666667 |
| 6339 | SCNN1D | 2.05E-14 | -4.6666667 |
| 2921 | CXCL3 | 2.51E-12 | -4.6829268 |
| 341640 | FREM2 | 1.56E-105 | -4.6923077 |
| 256126 | SYCE2 | 0.00049021 | -4.6923077 |
| 5860 | QDPR | 1.17E-53 | -4.6940299 |
| BGI_novel_G000264 | BGI_novel_G000264 | 2.37E-13 | -4.7121212 |
| BGI_novel_G001105 | BGI_novel_G001105 | 2.29E-17 | -4.7136364 |
| 5920 | RARRES3 | 3.19E-64 | -4.7154472 |
| 3373 | HYAL1 | 1.01E-14 | -4.7254902 |
| 9196 | KCNAB3 | 2.25E-14 | -4.7272727 |
| BGI_novel_G000316 | BGI_novel_G000316 | 3.81E-59 | -4.73125 |
| 6799 | SULT1A2 | 2.51E-06 | -4.7333333 |
| 51661 | FKBP7 | 1.13E-17 | -4.7391304 |
| 5275 | SERPINB13 | 8.73E-05 | -4.75 |
| 729359 | PLIN4 | 1.51E-14 | -4.75 |
| 375775 | PNPLA7 | 1.30E-06 | -4.75 |
| 3700 | ITIH4 | 1.00E-07 | -4.75 |
| 9963 | SLC23A1 | 0.00171138 | -4.75 |
| 285195 | SLC9A9 | 1.47E-09 | -4.75 |
| BGI_novel_G000129 | BGI_novel_G000129 | 1.48E-13 | -4.75 |
| 4256 | MGP | 2.37E-66 | -4.7875648 |
| 10451 | VAV3 | 1.46E-141 | -4.798913 |
| 647310 | TEX22 | 6.29E-08 | -4.8 |
| BGI_novel_G000138 | BGI_novel_G000138 | 0.1661948 | -4.8 |
| 80139 | ZNF703 | 1.50E-65 | -4.8266667 |
| 7052 | TGM2 | 9.21E-09 | -4.8333333 |
| 90019 | SYT8 | 3.28E-33 | -4.8536585 |
| 6623 | SNCG | 0 | -4.8630583 |
| 53335 | BCL11A | 3.50E-16 | -4.8823529 |
| 84734 | FAM167B | 1.36E-05 | -4.8888889 |
| 57593 | EBF4 | 2.27E-08 | -4.8947368 |
| 93659 | CGB5 | 0.00171138 | -4.9090909 |
| BGI_novel_G000692 | BGI_novel_G000692 | 0.01085394 | -4.9166667 |
| 100287284 | MANSC4 | 1.94E-29 | -4.9361702 |
| BGI_novel_G000875 | BGI_novel_G000875 | 2.36E-23 | -4.972973 |
| 11254 | SLC6A14 | 7.62E-37 | -5 |
| 152015 | ROPN1B | 0.00339824 | -5 |
| 3849 | KRT2 | 0.0893074 | -5 |
| 11251 | PTGDR2 | 0.00668258 | -5 |
| 117532 | TMC2 | 0.126212 | -5 |
| 339416 | ANKRD45 | 0.0893074 | -5 |
| 29906 | ST8SIA5 | 0.0893074 | -5 |
| 4135 | MAP6 | 0.00610434 | -5 |
| 395 | ARHGAP6 | 0.0246492 | -5 |
| 51267 | CLEC1A | 0.0123438 | -5 |
| 83937 | RASSF4 | 1.00E-11 | -5 |
| 166336 | PRICKLE2 | 0.00440746 | -5 |
| 85449 | KIAA1755 | 0.01557568 | -5 |
| 114898 | C1QTNF2 | 0.0893074 | -5 |
| 5284 | PIGR | 0.022553 | -5 |
| 126014 | OSCAR | 0.30275 | -5 |
| 4583 | MUC2 | 8.03E-06 | -5 |
| 202500 | TCTE1 | 0.30275 | -5 |
| 199786 | FAM129C | 0.1661948 | -5 |
| 10085 | EDIL3 | 0.00053709 | -5 |
| 374860 | ANKRD30B | 0.0331016 | -5 |
| 8302 | KLRC4 | 0.0472188 | -5 |
| 3751 | KCND2 | 0.00668258 | -5 |
| 10164 | CHST4 | 0.1661948 | -5 |
| 780776 | TVP23A | 0.0246492 | -5 |
| 284297 | SSC5D | 8.73E-05 | -5 |
| 53630 | BCO1 | 0.00339824 | -5 |
| 3860 | KRT13 | 0 | -5.004686 |
| 6511 | SLC1A6 | 0 | -5.0114286 |
| 9976 | CLEC2B | 7.77E-19 | -5.03125 |
| 9315 | NREP | 5.22E-150 | -5.0334572 |
| 26150 | RIBC2 | 6.89E-07 | -5.0666667 |
| 79679 | VTCN1 | 4.39E-59 | -5.076087 |
| 285386 | TPRG1 | 7.95E-57 | -5.1132075 |
| 117286 | CIB3 | 3.88E-08 | -5.1176471 |
| 347744 | C6orf52 | 0.022553 | -5.125 |
| 139728 | PNCK | 0.00053709 | -5.1666667 |
| 101927503 | LOC101927503 | 0.00339824 | -5.1666667 |
| BGI_novel_G000814 | BGI_novel_G000814 | 0.00049021 | -5.1666667 |
| 116729 | PPP1R27 | 0.056584 | -5.1666667 |
| 2323 | FLT3LG | 3.08E-13 | -5.175 |
| 83546 | RTBDN | 0.00358358 | -5.2 |
| 120376 | COLCA2 | 2.21E-07 | -5.2222222 |
| 10863 | ADAM28 | 8.66E-27 | -5.2307692 |
| BGI_novel_G001219 | BGI_novel_G001219 | 5.21E-17 | -5.2307692 |
| 1232 | CCR3 | 0.00339824 | -5.25 |
| 222663 | SCUBE3 | 1.56E-34 | -5.2857143 |
| 100316904 | SAP25 | 4.45E-06 | -5.3 |
| 5064 | PALM | 2.76E-28 | -5.3225806 |
| 4987 | OPRL1 | 0.00149511 | -5.3333333 |
| BGI_novel_G000757 | BGI_novel_G000757 | 2.39E-05 | -5.3333333 |
| 2619 | GAS1 | 2.30E-06 | -5.3333333 |
| 1230 | CCR1 | 0.00055697 | -5.3333333 |
| 1306 | COL15A1 | 2.42E-06 | -5.3333333 |
| 389895 | LOC389895 | 0.00187457 | -5.3333333 |
| BGI_novel_G000043 | BGI_novel_G000043 | 0.022553 | -5.3571429 |
| 5212 | VIT | 2.85E-26 | -5.3589744 |
| 2766 | GMPR | 5.65E-13 | -5.3846154 |
| 56147 | PCDHA1 | 1.54E-20 | -5.3846154 |
| 11093 | ADAMTS13 | 1.14E-09 | -5.4 |
| 4599 | MX1 | 3.29E-10 | -5.4 |
| 284339 | TMEM145 | 0.00096246 | -5.4 |
| 23475 | QPRT | 2.95E-17 | -5.4166667 |
| 151056 | PLB1 | 2.90E-22 | -5.4166667 |
| 1501 | CTNND2 | 2.89E-11 | -5.4285714 |
| 126006 | PCP2 | 0.00014713 | -5.45 |
| 7869 | SEMA3B | 4.81E-27 | -5.4583333 |
| 103344718 | HOTS | 0 | -5.4756396 |
| 6616 | SNAP25 | 0.022553 | -5.5 |
| 163255 | ZNF540 | 0.00339824 | -5.5 |
| 23630 | KCNE5 | 0.0472188 | -5.5 |
| 9938 | ARHGAP25 | 4.85E-05 | -5.5 |
| 150368 | FAM109B | 0.0123438 | -5.5 |
| 84514 | GHDC | 1.78E-14 | -5.5 |
| 5803 | PTPRZ1 | 1.05E-10 | -5.5 |
| 1520 | CTSS | 0.00096246 | -5.5 |
| 728392 | LOC728392 | 0.00053709 | -5.5 |
| 30061 | SLC40A1 | 1.82E-13 | -5.5454545 |
| 84290 | CAPNS2 | 2.42E-06 | -5.5625 |
| 5623 | PSPN | 0.00053709 | -5.5714286 |
| 245973 | ATP6V1C2 | 4.97E-93 | -5.5747126 |
| 83715 | ESPN | 7.29E-77 | -5.5846154 |
| BGI_novel_G000302 | BGI_novel_G000302 | 1.15E-09 | -5.6 |
| 83723 | FAM57B | 0.00096246 | -5.6 |
| 54504 | CPVL | 5.83E-13 | -5.6190476 |
| 2634 | GBP2 | 6.39E-230 | -5.6666667 |
| 51296 | SLC15A3 | 0.00339824 | -5.6666667 |
| 100996693 | LOC100996693 | 0.0893074 | -5.6666667 |
| 5409 | PNMT | 0.0472188 | -5.6666667 |
| 6707 | SPRR3 | 1.38E-82 | -5.6713781 |
| 4440 | MSI1 | 1.60E-29 | -5.6785714 |
| 8537 | BCAS1 | 5.39E-46 | -5.6829268 |
| 8820 | HESX1 | 4.85E-05 | -5.7 |
| 375704 | ENHO | 3.34E-12 | -5.7096774 |
| 6657 | SOX2 | 0 | -5.7134312 |
| 2045 | EPHA7 | 6.45E-19 | -5.7142857 |
| 11223 | MST1L | 3.32E-12 | -5.7142857 |
| 2001 | ELF5 | 0.0001638 | -5.75 |
| 129080 | EMID1 | 0.00053709 | -5.75 |
| 26471 | NUPR1 | 9.51E-18 | -5.7758621 |
| 92346 | C1orf105 | 1.47E-05 | -5.7894737 |
| BGI_novel_G000680 | BGI_novel_G000680 | 0.00055697 | -5.8 |
| 3882 | KRT32 | 1.07E-11 | -5.8 |
| 4056 | LTC4S | 5.31E-18 | -5.8101266 |
| 644943 | RASSF10 | 5.77E-13 | -5.8333333 |
| 200035 | NUDT17 | 0.00100611 | -5.8333333 |
| 135886 | WBSCR28 | 5.38E-14 | -5.8421053 |
| 733 | C8G | 0.00187457 | -5.875 |
| 4477 | MSMB | 4.45E-06 | -5.90625 |
| 4016 | LOXL1 | 4.98E-49 | -5.9464286 |
| BGI_novel_G000208 | BGI_novel_G000208 | 0.0213412 | -5.95 |
| 124975 | GGT6 | 1.66E-98 | -5.984127 |
| 1117 | CHI3L2 | 0.00055697 | -6 |
| 721 | C4B | 2.28E-07 | -6 |
| 338645 | LUZP2 | 4.02E-07 | -6 |
| 9568 | GABBR2 | 2.03E-08 | -6 |
| 83445 | GSG1 | 0.00102561 | -6 |
| BGI_novel_G000004 | BGI_novel_G000004 | 0.022553 | -6 |
| 2824 | GPM6B | 0.01085394 | -6 |
| BGI_novel_G000793 | BGI_novel_G000793 | 0.0454834 | -6 |
| 80258 | EFHC2 | 0.0406852 | -6 |
| 285313 | IGSF10 | 0.156618 | -6 |
| 4811 | NID1 | 0.00100611 | -6 |
| BGI_novel_G000169 | BGI_novel_G000169 | 0.0893074 | -6 |
| 4321 | MMP12 | 0.1661948 | -6 |
| 127670 | TEDDM1 | 0.0472188 | -6 |
| 158326 | FREM1 | 2.64E-05 | -6 |
| BGI_novel_G000107 | BGI_novel_G000107 | 0.0246492 | -6 |
| 362 | AQP5 | 0.1661948 | -6 |
| 84174 | SLA2 | 0.0893074 | -6 |
| BGI_novel_G000915 | BGI_novel_G000915 | 0.0246492 | -6 |
| 130589 | GALM | 0.0472188 | -6 |
| 116441 | TM4SF18 | 0.00096246 | -6 |
| 56105 | PCDHGA11 | 0.0123438 | -6 |
| 399671 | HEATR4 | 0.01273574 | -6 |
| BGI_novel_G000082 | BGI_novel_G000082 | 0.0454834 | -6 |
| 5158 | PDE6B | 0.01001342 | -6 |
| 85301 | COL27A1 | 2.53E-25 | -6 |
| BGI_novel_G001351 | BGI_novel_G001351 | 0.0472188 | -6 |
| 53353 | LRP1B | 1.11E-06 | -6 |
| 100526693 | ARPC4-TTLL3 | 5.83E-41 | -6.0175439 |
| 54873 | PALMD | 8.25E-42 | -6.047619 |
| BGI_novel_G001346 | BGI_novel_G001346 | 2.46E-06 | -6.047619 |
| 11155 | LDB3 | 1.12E-31 | -6.0526316 |
| 3623 | INHA | 1.09E-10 | -6.0555556 |
| 525 | ATP6V1B1 | 1.66E-15 | -6.1578947 |
| 130367 | SGPP2 | 1.50E-46 | -6.2083333 |
| 83690 | CRISPLD1 | 2.82E-43 | -6.2222222 |
| BGI_novel_G000585 | BGI_novel_G000585 | 1.29E-47 | -6.2337662 |
| 254173 | TTLL10 | 0.0001638 | -6.25 |
| 368 | ABCC6 | 0.00053709 | -6.25 |
| 643669 | CCER2 | 0.0123438 | -6.25 |
| 280 | AMY2B | 5.15E-17 | -6.2777778 |
| 5224 | PGAM2 | 8.06E-06 | -6.2857143 |
| 7166 | TPH1 | 0.0019058 | -6.3333333 |
| 8646 | CHRD | 3.31E-10 | -6.3333333 |
| 140766 | ADAMTS14 | 7.03E-08 | -6.3333333 |
| 143282 | FGFBP3 | 4.25E-26 | -6.375 |
| 80303 | EFHD1 | 5.38E-14 | -6.4 |
| 391322 | LOC391322 | 0.0472188 | -6.4 |
| 199699 | DAND5 | 0.0001638 | -6.4 |
| 3075 | CFH | 6.87E-40 | -6.4705882 |
| BGI_novel_G000433 | BGI_novel_G000433 | 3.00E-12 | -6.5 |
| 494513 | DFNB59 | 3.31E-10 | -6.5 |
| 196374 | KRT78 | 0.0019058 | -6.5 |
| 388135 | C15orf59 | 6.04E-07 | -6.5 |
| 924 | CD7 | 0.0019058 | -6.5 |
| 5657 | PRTN3 | 2.87E-13 | -6.5806452 |
| 283403 | C12orf80 | 0.00102561 | -6.6 |
| 64220 | STRA6 | 0 | -6.6 |
| 220963 | SLC16A9 | 7.97E-31 | -6.65 |
| 128229 | TSACC | 7.38E-07 | -6.6521739 |
| 4128 | MAOA | 5.24E-09 | -6.6666667 |
| 128178 | EDARADD | 4.88E-05 | -6.6666667 |
| 684 | BST2 | 2.00E-26 | -6.6896552 |
| 91653 | BOC | 1.36E-146 | -6.7222222 |
| 221416 | C6orf223 | 9.78E-11 | -6.75 |
| 4485 | MST1 | 9.30E-12 | -6.8 |
| 56241 | SUSD2 | 1.67E-194 | -6.8432836 |
| 26548 | ITGB1BP2 | 4.88E-05 | -6.8571429 |
| 2027 | ENO3 | 4.02E-07 | -6.8888889 |
| 89780 | WNT3A | 2.90E-69 | -6.9795918 |
| 56203 | LMOD3 | 0.00030044 | -7 |
| 9992 | KCNE2 | 0.00358358 | -7 |
| 7136 | TNNI2 | 0.00016107 | -7 |
| 54739 | XAF1 | 2.36E-08 | -7 |
| 55335 | NIPSNAP3B | 0.00027515 | -7 |
| BGI_novel_G000212 | BGI_novel_G000212 | 0.0893074 | -7 |
| 283571 | PROX2 | 0.00331906 | -7 |
| 3235 | HOXD9 | 0.0893074 | -7 |
| 161502 | CFAP161 | 0.1661948 | -7 |
| 2134 | EXTL1 | 0.00668258 | -7 |
| 284948 | SH2D6 | 0.0893074 | -7 |
| 64091 | POPDC2 | 0.0893074 | -7 |
| 479 | ATP12A | 0.00331906 | -7 |
| 5122 | PCSK1 | 0.00100611 | -7 |
| 128653 | C20orf141 | 0.00358358 | -7 |
| 343505 | NBPF7 | 0.0454834 | -7 |
| 93979 | CPA5 | 0.0893074 | -7 |
| 644150 | WIPF3 | 0.00668258 | -7 |
| 7113 | TMPRSS2 | 0.00652474 | -7 |
| 753 | LDLRAD4 | 2.40E-05 | -7 |
| 2268 | FGR | 0.0246492 | -7 |
| BGI_novel_G000001 | BGI_novel_G000001 | 8.52E-14 | -7.2222222 |
| 23302 | WSCD1 | 2.11E-95 | -7.3125 |
| 1056 | CEL | 2.99E-121 | -7.3173077 |
| 6691 | SPINK2 | 0.00358358 | -7.3333333 |
| 57214 | CEMIP | 4.86E-46 | -7.3333333 |
| 286 | ANK1 | 5.35E-05 | -7.3333333 |
| 116372 | LYPD1 | 6.37E-15 | -7.4285714 |
| 7318 | UBA7 | 4.50E-22 | -7.4545455 |
| 3293 | HSD17B3 | 3.08E-36 | -7.4761905 |
| 100528032 | KLRC4-KLRK1 | 0.00668258 | -7.5 |
| 1082 | CGB3 | 0.00331906 | -7.5 |
| 1960 | EGR3 | 2.42E-30 | -7.5 |
| 2702 | GJA5 | 1.56E-08 | -7.5 |
| 3784 | KCNQ1 | 1.19E-18 | -7.5454545 |
| 158511 | CSAG1 | 0.00100611 | -7.625 |
| 84696 | ABHD1 | 0.0019058 | -7.6666667 |
| 1482 | NKX2-5 | 1.84E-08 | -7.75 |
| 25837 | RAB26 | 4.21E-286 | -7.764526 |
| 55753 | OGDHL | 5.47E-14 | -7.8333333 |
| 4222 | MEOX1 | 3.24E-32 | -7.8461538 |
| 4640 | MYO1A | 0.30275 | -8 |
| 3561 | IL2RG | 0.0893074 | -8 |
| 1589 | CYP21A2 | 0.00358358 | -8 |
| 283152 | CCDC153 | 0.0019058 | -8 |
| BGI_novel_G000610 | BGI_novel_G000610 | 0.0893074 | -8 |
| BGI_novel_G000396 | BGI_novel_G000396 | 0.00668258 | -8 |
| BGI_novel_G000294 | BGI_novel_G000294 | 0.0893074 | -8 |
| 9543 | IGDCC3 | 2.03E-08 | -8 |
| 84210 | ANKRD20A1 | 5.80E-12 | -8 |
| 579 | NKX3-2 | 4.55E-05 | -8 |
| 10661 | KLF1 | 0.0893074 | -8 |
| 57463 | AMIGO1 | 0.01273574 | -8 |
| 163782 | KANK4 | 0.0019058 | -8 |
| 100288797 | TMEM239 | 0.01273574 | -8 |
| 256158 | HMCN2 | 8.74E-11 | -8 |
| 283869 | NPW | 0.0472188 | -8 |
| BGI_novel_G001066 | BGI_novel_G001066 | 0.00331906 | -8 |
| 55584 | CHRNA9 | 4.50E-22 | -8.0769231 |
| 1299 | COL9A3 | 1.82E-47 | -8.09375 |
| 6515 | SLC2A3 | 2.04E-146 | -8.1612903 |
| 10628 | TXNIP | 0 | -8.3303199 |
| 54847 | SIDT1 | 9.24E-10 | -8.3333333 |
| 389058 | SP5 | 1.56E-08 | -8.3333333 |
| 374973 | TEX38 | 0.0246492 | -8.3333333 |
| 6948 | TCN2 | 8.59E-05 | -8.3333333 |
| 8214 | DGCR6 | 3.13E-11 | -8.3846154 |
| 342527 | SMTNL2 | 1.67E-07 | -8.4 |
| 130497 | OSR1 | 2.79E-09 | -8.4285714 |
| 339403 | RXFP4 | 0.0246492 | -8.5 |
| 100128327 | TRAPPC3L | 0.0246492 | -8.5 |
| 1749 | DLX5 | 5.51E-104 | -8.6724138 |
| 7134 | TNNC1 | 2.97E-13 | -8.7241379 |
| 51252 | FAM178B | 4.26E-15 | -8.7916667 |
| 100129792 | CCDC152 | 0.0016782 | -9 |
| 158297 | SAXO1 | 0.0472188 | -9 |
| 9508 | ADAMTS3 | 8.59E-05 | -9 |
| 55897 | MESP1 | 0.01273574 | -9 |
| 2687 | GGT5 | 0.00652474 | -9 |
| 100129583 | FAM47E | 1.56E-08 | -9 |
| 123872 | DNAAF1 | 0.0246492 | -9 |
| 134548 | SOWAHA | 0.00358358 | -9 |
| 1538 | CYLC1 | 2.96E-08 | -9 |
| 79949 | PLEKHS1 | 0.022553 | -9 |
| 254827 | NAALADL2 | 1.76E-35 | -9.2 |
| 6678 | SPARC | 3.95E-11 | -9.25 |
| 7351 | UCP2 | 2.45E-32 | -9.2962963 |
| 132430 | PABPC4L | 1.19E-12 | -9.3333333 |
| 8794 | TNFRSF10C | 9.27E-07 | -9.4 |
| 84870 | RSPO3 | 2.90E-14 | -9.5 |
| 124590 | USH1G | 2.41E-17 | -9.5 |
| 284194 | LGALS9B | 1.36E-82 | -9.5692308 |
| 5608 | MAP2K6 | 1.24E-84 | -9.625 |
| 10321 | CRISP3 | 2.43E-18 | -9.7 |
| 441519 | CT45A3 | 4.78E-07 | -9.75 |
| 7941 | PLA2G7 | 1.19E-13 | -9.7777778 |
| 4988 | OPRM1 | 8.59E-05 | -10 |
| 50853 | VILL | 0.0016782 | -10 |
| 344905 | ATP13A5 | 0.00021081 | -10 |
| 260293 | CYP4X1 | 0.01273574 | -10 |
| 53822 | FXYD7 | 0.00331906 | -10 |
| 51733 | UPB1 | 0.01273574 | -10 |
| 1496 | CTNNA2 | 0.0019058 | -10 |
| 22953 | P2RX2 | 1.07E-64 | -10.319149 |
| 63970 | TP53AIP1 | 0.00030044 | -10.5 |
| 3965 | LGALS9 | 8.26E-104 | -10.623188 |
| 6947 | TCN1 | 8.62E-11 | -11 |
| 3212 | HOXB2 | 0.0246492 | -11 |
| 55259 | CASC1 | 8.21E-13 | -11 |
| 84457 | PHYHIPL | 0.00021081 | -11 |
| 132851 | SPATA4 | 0.00331906 | -11 |
| 8764 | TNFRSF14 | 8.62E-11 | -11 |
| 56892 | C8orf4 | 1.06E-71 | -11.046512 |
| 23428 | SLC7A8 | 2.59E-21 | -11.428571 |
| 389383 | CLPSL2 | 3.30E-08 | -11.578947 |
| 165679 | SPTSSB | 3.30E-08 | -12 |
| 1577 | CYP3A5 | 0.0472188 | -12 |
| 3586 | IL10 | 0.00469836 | -12 |
| 10841 | FTCD | 0.00652474 | -12 |
| 653808 | ZG16 | 0.01273574 | -12 |
| 8928 | FOXH1 | 1.94E-05 | -12 |
| 10346 | TRIM22 | 1.46E-24 | -12.142857 |
| 10161 | LPAR6 | 1.82E-45 | -12.176471 |
| 1580 | CYP4B1 | 1.99E-12 | -12.6 |
| 5764 | PTN | 1.16E-10 | -12.8 |
| 3236 | HOXD10 | 0.00652474 | -13 |
| 26011 | TENM4 | 6.08E-46 | -13 |
| 4151 | MB | 3.83E-22 | -13.055556 |
| 653361 | NCF1 | 0.00010477 | -13.5 |
| BGI_novel_G000666 | BGI_novel_G000666 | 2.23E-97 | -13.536232 |
| 11272 | PRR4 | 1.49E-06 | -14 |
| 7805 | LAPTM5 | 1.29E-06 | -14 |
| 116844 | LRG1 | 2.66E-35 | -14.333333 |
| 10964 | IFI44L | 2.27E-25 | -14.333333 |
| 83896 | KRTAP3-1 | 1.29E-06 | -14.375 |
| 55503 | TRPV6 | 1.68E-08 | -14.5 |
| 79783 | SUGCT | 8.89E-68 | -14.981818 |
| 4613 | MYCN | 5.05E-09 | -15 |
| 133690 | CAPSL | 1.64E-07 | -15 |
| 2128 | EVX1 | 7.37E-05 | -15 |
| 23145 | SSPO | 7.45E-11 | -15 |
| 3624 | INHBA | 1.17E-93 | -16 |
| 3037 | HAS2 | 1.85E-21 | -16.5 |
| 11166 | SOX21 | 3.49E-187 | -16.560976 |
| 29970 | SCHIP1 | 3.71E-58 | -16.625 |
| 90865 | IL33 | 5.19E-05 | -17 |
| 163688 | CALML6 | 0.00042269 | -17 |
| 10581 | IFITM2 | 7.69E-197 | -17.377778 |
| 25984 | KRT23 | 2.17E-239 | -17.96 |
| 1307 | COL16A1 | 2.82E-10 | -18 |
| 1959 | EGR2 | 6.19E-118 | -18.043478 |
| 3371 | TNC | 0 | -18.458333 |
| 6783 | SULT1E1 | 6.69E-27 | -18.625 |
| 23539 | SLC16A8 | 8.19E-08 | -19 |
| 3852 | KRT5 | 4.09E-34 | -19.25 |
| 51806 | CALML5 | 6.21E-06 | -19.666667 |
| 1907 | EDN2 | 1.64E-07 | -19.666667 |
| 27098 | CLUL1 | 2.55E-06 | -20 |
| 7475 | WNT6 | 0.00042269 | -20 |
| 9220 | TIAF1 | 0 | -20.552381 |
| 401944 | LDLRAD2 | 0 | -20.925 |
| 199974 | CYP4Z1 | 5.19E-05 | -22 |
| 79927 | FAM110D | 0.00010477 | -25 |
| 53826 | FXYD6 | 2.56E-05 | -25 |
| 155465 | AGR3 | 3.30E-15 | -27.428571 |
| 3488 | IGFBP5 | 1.36E-298 | -29.466667 |
| 340596 | LHFPL1 | 3.05E-06 | -30 |
| 8519 | IFITM1 | 0 | -32.652318 |
| 4004 | LMO1 | 8.45E-08 | -33.5 |
| 26659 | OR7A5 | 4.68E-09 | -35 |
| BGI_novel_G000196 | BGI_novel_G000196 | 5.75E-47 | -36.538462 |
| 6781 | STC1 | 0 | -48.904762 |
| BGI_novel_G001261 | BGI_novel_G001261 | 2.89E-189 | -96.444444 |
